# Variation in the Fitness Effects of Mutations with Population Density and Size in *Escherichia coli*

**DOI:** 10.1371/journal.pone.0105369

**Published:** 2014-08-14

**Authors:** Huansheng Cao, Kevin Butler, Mithi Hossain, James D. Lewis

**Affiliations:** Louis Calder Center–Biological Field Station and Department of Biological Sciences, Fordham University, Armonk, New York, United States of America; Fred Hutchinson Cancer Research Center, United States of America

## Abstract

The fitness effects of mutations are context specific and depend on both external (e.g., environment) and internal (e.g., cellular stress, genetic background) factors. The influence of population size and density on fitness effects are unknown, despite the central role population size plays in the supply and fixation of mutations. We addressed this issue by comparing the fitness of 92 Keio strains (*Escherichia coli* K12 single gene knockouts) at comparatively high (1.2×10^7^ CFUs/mL) and low (2.5×10^2^ CFUs/mL) densities, which also differed in population size (high: 1.2×10^8^; low: 1.25×10^3^). Twenty-eight gene deletions (30%) exhibited a fitness difference, ranging from 5 to 174% (median: 35%), between the high and low densities. Our analyses suggest this variation among gene deletions in fitness responses reflected in part both gene orientation and function, of the gene properties we examined (genomic position, length, orientation, and function). Although we could not determine the relative effects of population density and size, our results suggest fitness effects of mutations vary with these two factors, and this variation is gene-specific. Besides being a mechanism for density-dependent selection (*r*-*K* selection), the dependence of fitness effects on population density and size has implications for any population that varies in size over time, including populations undergoing evolutionary rescue, species invasions into novel habitats, and cancer progression and metastasis. Further, combined with recent advances in understanding the roles of other context-specific factors in the fitness effects of mutations, our results will help address theoretical and applied biological questions more realistically.

## Introduction

Mutations are the ultimate source of genetic variation within populations and the cause of antibiotic resistance, pathogen virulence, and many human diseases. The fixation probability of mutations in populations reflects their fitness effects, the sizes of the populations in which they arise, and other factors [1], [2]. Therefore, predicting the fixation of mutations and the evolutionary trajectories of populations requires accurate estimates of each of these factors, as well as an understanding of the relationships among them. Empirical work using model organisms has established that the fitness effects of a given mutation are context specific. For example, both external environment, such as medium and temperature [3], [4], and internal factors, including growth rate [5], cellular stress state [6] and genetic background (epistasis) [7], influence the fitness effects of mutations. Yet, little is known about whether the fitness effects of mutations vary with population size or density, despite the large variation these exhibit in natural populations.

Several lines of evidence suggest population density or size, which often covary, may influence the fitness effects of mutations. An early study in *Drosophila melanogaster* found that variance in offspring viability changes with parental population density [4]. Recently, some small *Escherichia coli* populations were found to exceed large populations in fitness gains in experimental evolution [8]. Marx reported that a *Methylobacterium extorquens* population was more fit at low than high density [9]. However, the identities of the mutations involved in these studies were not established. Therefore, it is currently unknown whether the density- or size-dependence of fitness effects is a general property of mutations. This issue seems to have been ‘overlooked’, probably because the fitness effect of a given mutation has been assumed to be independent of population density or size [10], [11].

It is important, however, to understand this relationship, as nearly all natural populations are finite in size [12], [13]. More importantly, population sizes and densities often fluctuate with environments and population life history [14]–[17]. Few studies have addressed the influences of population size or density on the fitness effects of mutations, even in model organisms. Here, we addressed this question by comparing the fitness of 92 Keio knockout strains [18] between comparatively high (1.2×10^7^ CFUs/mL) and low density (2.5×10^2^ CFUs/mL) in minimal medium. As population size also differed between treatments, our study addressed the combined effects of changes in population density and size. Each Keio strain had a specifically defined deletion of a single non-essential gene.

## Materials and Methods

### Keio knockout strains

**Figure 1:**
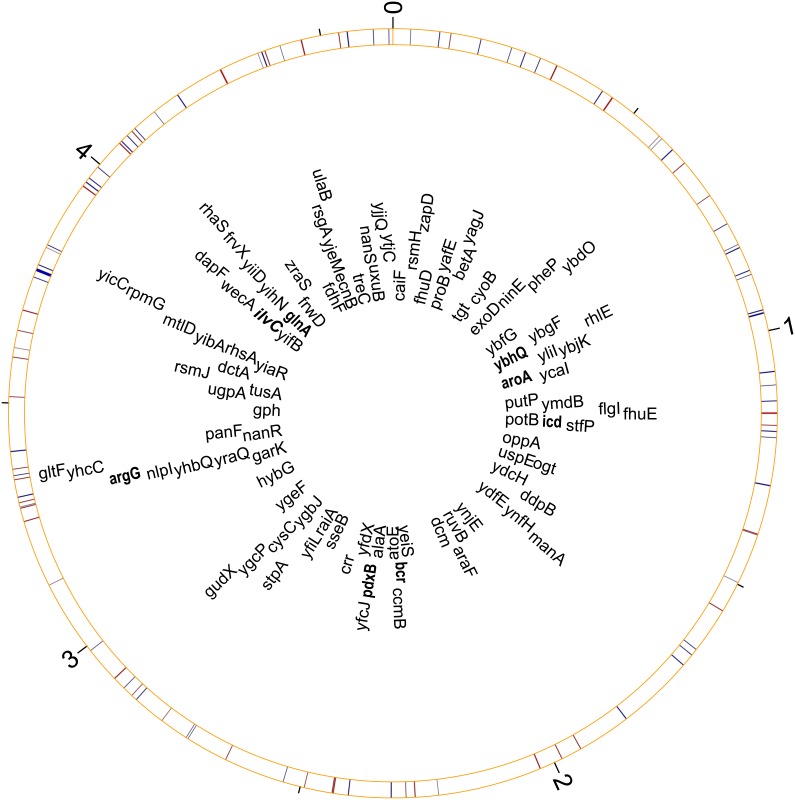
The characteristics of the deleted genes used in this study. This Circos map was plotted based on the genome of *Escherichia coli* K12 MG1655, which is the relative of the progenitor of all Keio knockouts, BW25113. The gene labels in bold indicate the eight mutants tested but not included in final fitness analyses due to slow growth during conditioning. Blue bars indicate the genes are on the coding strand and red bars the template strand. The thickness of the bars represents gene length; the numbers on the outer ticks shows the scale of the genome in megabases.

All mutants used in this study were selected from the Keio collection, which is a set of *E. coli* knockouts [18]. All the knockouts are generated from a common progenitor, *E. coli* K-12 BW25113; and each mutant has a single unique non-essential gene replaced in frame with a kanamycin resistance cassette through homologous recombination [18]. We randomly picked 100 out of the 3816 strains of the collection available at the *Coli* Genetic Stock Center (CGSC) at Yale University (Table S1). Each strain was colony purified upon receipt and stored in 15% glycerol stocks at −80°C. These deleted genes are randomly distributed on the wildtype genome (Figure 1). Strain JW1605-2, in which *manA* is deleted in frame [18], was used as a reference in fitness assays of the other Keio strains, following [19]. *manA* deletion abolishes mannose utilization capability in the mutant [20], causing it to appear red on tetrazolium mannose (TM) indicator plates. This allowed us to distinguish it from the white wildtype colonies on TM plates used for enumerating each competitor in fitness assays.

### Fitness assays

We assayed the fitnesses of the Keio strains in competitions against the common competitor JW1605-2 at high and low density in batch culture. The high density treatment (High) was started at 1.2×10^7^ CFUs/mL, which approximated the size of free-living *E. coli* populations [21] and is the starting size commonly used in *E. coli* experimental evolution [22], [23]. The low density treatment (Low) started at 2.5×10^2^ CFUs/mL, following Perfeito et al. [24]. These two treatments gave a ∼5×10^4^-fold difference in density.

The fitness assays were adapted from Elena et al [19]. Briefly, each strain of interest, as well as JW1605-2, were revived from glycerol stocks in LB and acclimated to similar physiological states in MOPS Minimal medium [25] (Teknova, Hollister, CA, USA) supplemented with 0.2% glucose at 37°C and 120 rpm for 24 h. We then mixed both competitors at a 1∶1 ratio for competition in the High and Low treatments, with ten replicates for each treatment. For the High competitions, 50 µL of each competitor (ca. 6×10^7^ CFUs) was added to 9.9 mL of fresh MOPS medium. For the Low competitions, 150 µL of 2×10^−6^ dilution of each competitor (ca. 6.25×10^2^ CFUs) was added to 5 mL of MOPS medium [26]. These two volumes allowed both populations to reach similar carrying capacity during the growth cycle and to stay in the stationary phase for similar amounts of time (ca. 2 h) (Figure S1). During each growth cycle, the populations went through about 6.7 generations (doublings) in the High treatment and 25 generations in the Low treatment. To eliminate the difference in number of generations, we ran the competitions in the High treatment for four successive growth cycles (12 h each cycle). This way, cells spent similar time in stationary phase as those in the Low treatment (Figure S1), and went through about 27 generations of growth, which was comparable to the 25 generations in the Low treatment.

We enumerated each competitor at the start and end of the competition assays by spreading a sample of the culture on TM plates. Finally, the relative fitness (*W*) of each strain against the common competitor JW1605-2 was standardized against the common progenitor JW25113, by multiplying the mean fitness of JW1605-2 against JW25113, as previously described [19]. This approach will generate the fitnesses of the Keio strains against the original wildtype. It may systematically change the original relative fitnesses in the two treatments but not alter the comparisons between the two treatments. We also calculated the fold change of fitness between the two treatments using the mean fitness as follows:
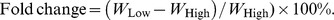



### Functional classes of the deleted genes in the Keio strains

As each mutant used in this study has a single defined gene deleted, we grouped these genes into functional classes based on the Clusters of Orthologous Groups of proteins (COGs) [27], in order to analyze the relationship between gene function and fitness responses to the two treatments.

### Statistical Analyses

Our design took into account the possibilities of both type I and II statistical errors associated with calling each fitness significantly different than that of the wildtype. To minimize type I error, we increased the replicate number to 10 from the required eight, based on a power test in a pilot study. To check for type II error, we repeated the assays (also with 10 replicates) for the common competitor in each treatment. The results from the pooled 20 replicates were not different from those obtained with the first 10 replicates. Statistical tests were performed with R 3.02 [28]. The fitness of each strain was compared between the two treatments using paired t-tests (*α* = 0.05). In cases where the normality requirement was not met, we used paired Wilcoxon signed-rank tests. The distribution of the fitness effects for all gene deletions were tested for normality for each treatment using the Shapiro-Wilk test and were compared between treatments using the Kolmogorov-Smirnov test.

## Results

### Fitness analyses for the Keio strains

We initially selected a total of 100 Keio strains (Table S1). However, eight strains (Table S1 and Figure 1) had low turbidity (OD_600_<0.2) after 48 h in conditioning, so we excluded them from subsequent analyses, resulting in 92 strains being used to assess the proportion of strains that responded differentially to the population treatments. Four other strains (JW0732-1, JW1327-1, JW1849-2, and JW4122-3) were driven extinct in all replicates by the common competitor JW1605-2 in both treatments, and one strain (JW3496-1) was driven extinct in six replicates in the Low treatment. Accordingly, we did not include these five strains in our fitness calculations, leaving a total of 87 strains used for these calculations (Table S1 and Figure S2).

### Frequency, magnitude and distribution of fitness responses

**Figure 2:**
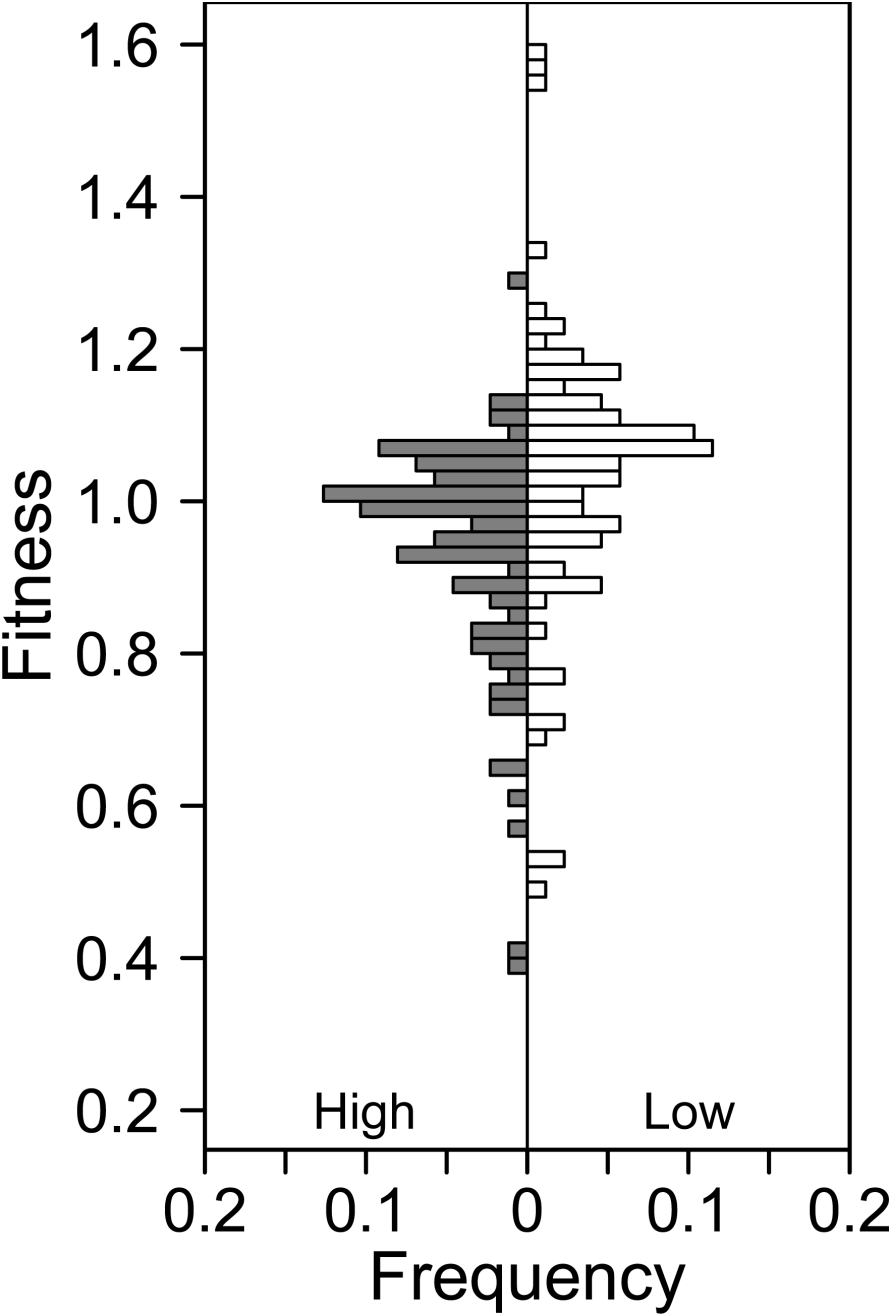
The fitness distribution of the studied Keio strains at in the High and Low treatments.

**Figure 3:**
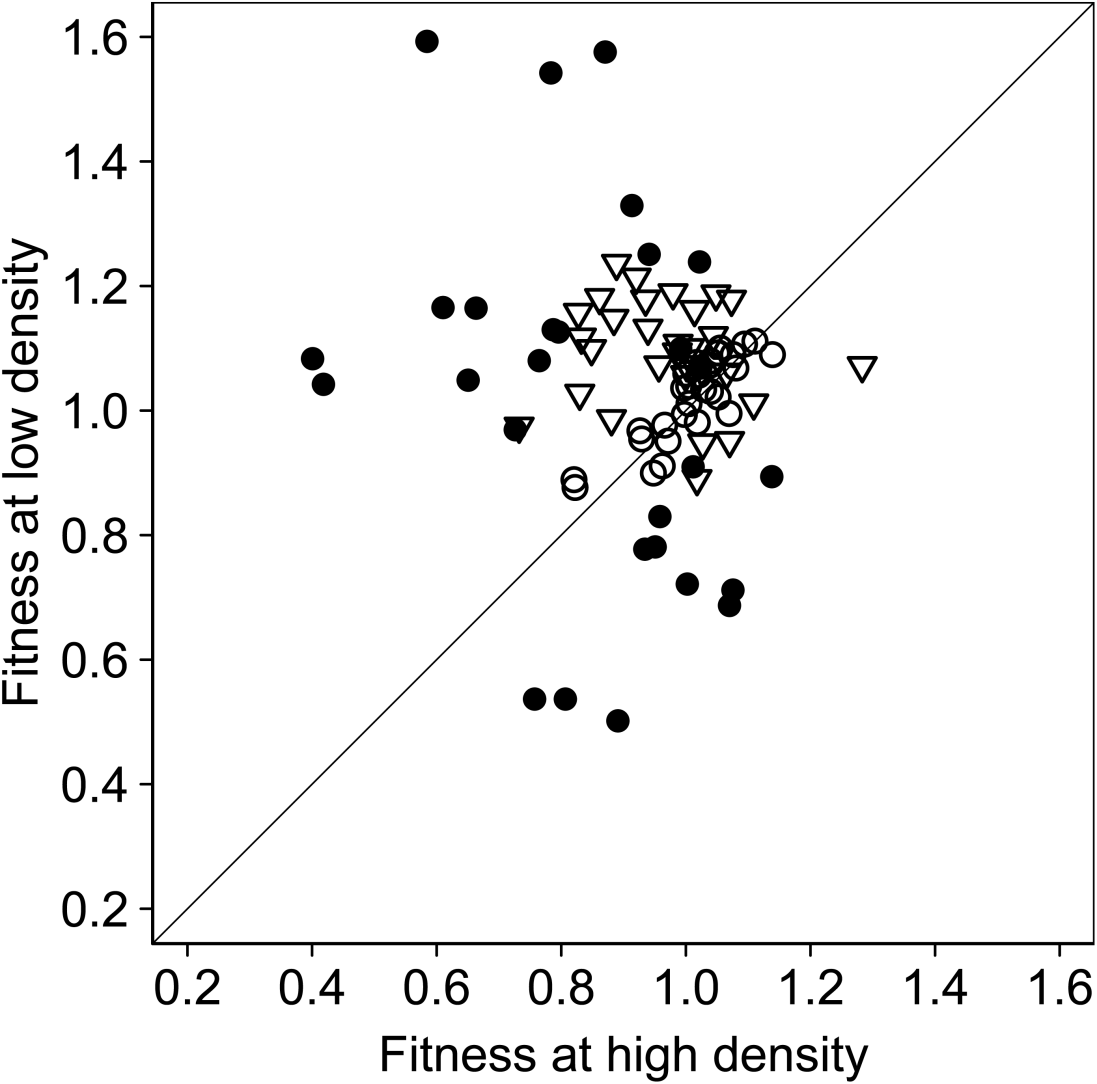
The fitness change in the studied Keio strains between in the High and Low treatments. The changes were either significantly different (filled circles), equivalent with high standard deviation (open triangles), or equivalent with low standard deviation (open circles).

For the 87 Keio strains where we could calculate fitness values (Figure S2), the frequency and proportion of the fitnesses of the gene knockouts differed between the High and Low treatments (Chi-square test, *χ* ^2^ = 18.2, df = 2, *P*<0.001). The proportions of beneficial, deleterious and neutral deletions were 13, 39 and 48% in the High treatment, compared with 34, 19 and 47% in the Low treatment, respectively (Figure 2). To assess whether there was a treatment effect on the fitness of a given mutant, we measured the quantitative change in the fitness for each strain between the High and Low treatments. Twenty-eight (30%) of the deletions differed in fitness between treatments (Paired t-tests or Wilcoxon tests, *P*<0.05; Figure 3). The fitness differences ranged from 5% to 174%, with a median of 35%, among these 28 strains (Figure S3 and Table S2). Seventeen (61%) of the 28 strains exhibited increased fitness in the Low treatment compared with the High treatment, while the remaining 11 (39%) strains had decreased fitness. Of the other 64 strains, 38% had similar fitnesses with low standard variation between treatments (Paired t-tests *P*>0.05), while 32% had similar fitnesses but with high standard variations (Wilcoxon tests, *P*>0.05; Figure 3).

Accordingly, mean (±1 SD) fitnesses in the High (0.94±0.15) and Low (1.04±0.18) treatments differed across deletions (Wilcoxon test, *W* = 2173, *P*<0.001). The ranges of fitness also were higher and broader in the Low treatment, as the ranges were 0.4∼1.3 in the High treatment and 0.5 ∼1.6 in the Low treatment (Kolmogorov-Smirnov test, *D* = 0.4023, *P*<0.01; Figure 2). We also examined the shape of the distribution of fitness effects in each treatment. Both distributions exhibited non-normal distributions (Shapiro-Wilk test, both *P*<0.01; Figure 2). The distribution in the High treatment was left skewed (*g*_1_ = −1.3) and platykurtic (*g*_2_ = 2.4). The fitness distribution in the Low treatment was also platykurtic (*g*_2_ = 2.4), but was less left skewed (*g*_1_ = −0.2).

### Gene characteristics associated with fitness responses

The Keio knockouts in this study were randomly selected (Table S1 and Figure 1). Results presented in earlier sections suggest that not all genes had the same fitness change between the treatments. To assess whether fitness responses were gene-specific, we performed a two-way ANOVA with square root transformed fitness values for treatment and gene identity. The ANOVA results were consistent with our other results, indicating that the treatment dependence of fitness effects varied with gene identity (*P*<0.01 for the interaction between treatment and gene identity on fitness values).

To further explore what gene characteristics were associated with this gene-specific response, we assessed the effects of genomic position, length, and orientation (coding vs. template strand) (Figure 1). For each treatment, we first performed Pearson’s correlation between fitness and the position (start or end) or length of the deleted genes, but found no strong correlations (*r* between −0.14 and 0.10 in all cases). For gene orientation, we split fitness into two orientation groups and compared them using a Kolmogorov-Smirnov test; based on this test, fitness significantly differed between orientation groups in the High (*D* = 0.360, *P*<0.01) but not in the Low (*D* = 0.189, *P*>0.05) treatment.

We also considered the effect of gene function, but our dataset precluded such analysis because some functional groups had few genes (Table 1). Further, gene function and orientation are tied together for a given gene and cannot be crossed in statistical tests. Therefore, we could not quantify the separate roles of gene orientation and function in gene-specific fitness changes. Nevertheless, descriptive statistics suggest that the treatment dependence of fitness effects varied with gene function. Specifically, the gene deletions examined in this study spanned 18 functional groups; of these, 13 groups included genes that exhibited treatment-dependent fitness (Table 1). Functional groups involved in information storage and processing, and cellular processes and signaling, disproportionately accounted for treatment-dependent genes. In contrast, genes from the metabolism group exhibited limited treatment-dependent fitness change. For example, energy production (C), coenzyme transport and metabolism (H), lipid metabolism (I), and secondary metabolite metabolism (Q) exhibited no clear treatment-dependent fitness change. Interestingly, several genes of unknown function also exhibited treatment-dependent fitness changes, suggesting these genes perform critical, though undiscovered, functions.

**Table 1:** The functional Clusters of Orthologous Groups (COGs) of the deleted genes in all 100 Keio strains studied and the proportions of genes showing treatment-dependent fitness difference in each functional group.

	COG code	Total number	Treatment-dependent number (fraction)^a^	Treatment-independent number (fraction)
Information storage and processing	J	4	3 (75%)	1 (25%)
	K	9	4 (44%)	5 (56%)
	L	6	2 (33%)	4 (67%)
Cellular processes and signaling	D	1	1 (100%)	0
	V	2	1 (50%)	1 (50%)
	T	3	1 (33%)	2 (67%)
	M	6	2 (33%)	4 (67%)
	N	2	2 (100%)	0
	O	4	1 (25%)	3 (75%)
Metabolism	C	5	0	5 (100%)
	G	17	3 (18%)	14 (82%)
	E	18	1 (6%)	17 (94%)
	H	4	0	4 (100%)
	I	2	0	2 (100%)
	P	8	1 (13%)	7 (87%)
	Q	2	0	2 (100%)
Poorly characterized	R	23	0	23 (100%)
	S	9	5 (56%)	4 (44%)

a two genes are dual and triple functional.

## Discussion

Intra-population competition plays a fundamental role in effecting natural selection in Darwinian evolution [29]. However, the fitness effects of mutations have long been assumed to be independent of population dynamics, such as density and size [10], [11]. Here we provide the first systematic evidence that fitness effects of mutations vary with population density or size. Of 92 defined single gene deletions in the Keio strains we tested, about 30% exhibited significant differences in fitness between the Low and High treatments. This proportion may be an underestimation, as the significance of difference for some strains was affected by large standard variation (Figures 3 and S2), as in *Drosophila* [4]. Our results build on previous research that found that small *E. coli* populations exceed large populations in fitness gains after 500 generations [8]. Together, these results suggest that fitness effects in *E. coli* depend on population size or density. Further, our results suggest that the effect is gene-specific and may reflect both gene function and orientation.

One potential confounding factor is differences in the amount of stationary phase time spent by populations in the two treatments. To minimize this effect, we did transfers for the High populations every 12 h, such that both treatments spent similar time (about two hours) after reaching carrying capacity (Figure S1). Also, it has been shown that the death rate during the stationary phase is not a significant component of overall fitness in batch cultures [30]. Therefore, we expect the effect of their time in stationary phase to be minimal. Another potential factor affecting fitness differences between the treatments is the culture volumes used in the two treatments. This effect is likely to be minimal, though, because the two-fold difference in volume (5 mL vs. 10 mL) would translate into a difference of one generation, compared with 25 elapsed generations during competition in the Low treatment. Additional potential confounding factors are long lag phase and high concentrations of glucose in Low populations compared with High populations. Our approaches of conditioning competitors to similar physiological states and reducing culture volumes in the Low treatment would help minimize the effects of these two potential factors, as the fitness estimates were based only on the initial and final population density. Indeed, High and Low treatment carrying capacities were similar (Figure S1), so the effect of the difference in glucose would be expected to be minimal, as in other studies establishing populations with different inocula [8], [24].

The treatment-dependence of fitness response was common among gene deletions, but not universal, in our study. Unlike studies on the fitness effects of anonymous mutations (e.g., [19], [24]), genomic characteristics of the tested genes in our study are well annotated and their functions have been either experimentally identified or inferred informatically (Table S1) [18]. This information enabled us to link treatment-dependent fitness response to physical gene characteristics and function. Our results suggest that gene-specific fitness responses were affected by both gene orientation and gene function, but gene locus and gene length did not have a clear effect. The role of gene orientation was unexpected but not surprising, as it is closely associated with gene expression and DNA replication [31], [32]. Our results also suggest that fitness responses varied among functional classes, as treatment effects were observed in only 13 of the 18 classes (Tables 1 and S2), and were not uniformly distributed across these 13 classes; carbohydrate metabolism, transcription, translation, and amino acid metabolism accounted for more treatment*-*dependent effects than the other classes combined. However, we were unable to estimate the relative contributions of gene orientation and gene function, not only because they are tied together for each gene, but also because orientation is also determined by function [33]. Nonetheless, our study using specifically defined mutations not only suggests that fitness effects of mutations often vary with population density or size, but that such dependence may vary with gene orientation and function.

Our finding that mutational fitness effects vary with population density provides a context for the fitness differences between high and low density observed in previous studies. In addition to effects of density observed in *Drosophila* [4] and evolved *E. coli* populations [8], Marx also observed fitness varied with density in one of his eight *Methylobacterium extorquens* AM1 populations [9]. At the molecular level, two genes, *yhiD* and *hdeD*, of the acid resistance mechanism were only expressed at high population density (>10^8^ CFUs per ml) [34]. Taken together with our establishment of the relationship between fitness effects of mutations and population density, these findings provide a mechanism for density-dependent (*r-K*) selection and trade-offs in life history traits [35].

Still unresolved, though, is the exact basis for why mutational fitness effects varied with population density or size. One possibility is quorum sensing; in this scenario, mutations at different densities cause differences in the production or dissemination of autoinducer signals [36], leading to the observed differences in fitness. Alternatively, if having a mutation acts as a stress that inflicts heat shock response, as in *Caenorhabditis elegans* [6], it would lead to degradation of ribosomes [37] or trigger protein quality control [38]. If ribosome degradation is slow, it would cause density-dependent growth inhibition and, thus, density-dependent fitness, similar to the inoculum effect of antibiotic resistance [37]. Whatever the mechanisms may be for the density-dependence of fitness effects, they have to account for the observations that such dependence is also gene specific.

In summary, our results suggest that fitness effects of mutations vary with population density and size, and these variations are gene specific. These results are consistent with previous studies, which have found that fitness effects of mutations are context dependent [3], [4], [6], [7]. However, our results are the first to suggest that the fitness effect of specific mutations may vary with population density or size. This variation in fitness effects has critical bearing on a wide range of theoretical and applied fields of biology, as nearly all natural populations have finite sizes that vary over time. Our results may be particularly important in understanding the fitness effects of mutations in small populations, such as the evolutionary rescue of endangered populations, invasions of species, proliferation of pathogenic populations, and cancer progression and metastasis. Coupled with recent advances in understanding the roles of other context-specific factors in the fitness effects of mutations, our results will help address biological questions more realistically.

## Supporting Information

Figure S1 **The growth curve of Keio progenitor BW25113 in the High and Low treatments over a 24-h cycle.** The orange vertical line indicates the timing of transfer to next growth cycle in large populations. Orange: High treatment; blue: Low treatment.
(TIF)

Figure S2 **The fitness of all 87 Keio strains in the High and Low treatments.** Error bars = 1 SD.
(TIF)

Figure S3 **The fitness change between the High and Low treatments in the 28 treatment-responsive Keio strains.** Gray bars: strains with deletions exhibiting opposing responses between the High and Low treatments; white bars: strains with deletions neutral in one treatment but non-neutral in the other; black bars: strains with deletions non-neutral in the same direction in both treatments.
(TIF)

Table S1 **The 100 Keio strains used in this study.**
(DOCX)

Table S2 **The functions of the deleted genes in the Keio strains showing population density-dependent** **fitness difference.**
(DOCX)
