# Supplementary figures and images for: Variation in the Fitness Effects of Mutations with Population Density and Size in Escherichia coli
Source: PLoS One. 2014 Aug 14;9(8):e105369. doi: 10.1371/journal.pone.0105369 (PMC4133409; doi:10.1371/journal.pone.0105369)

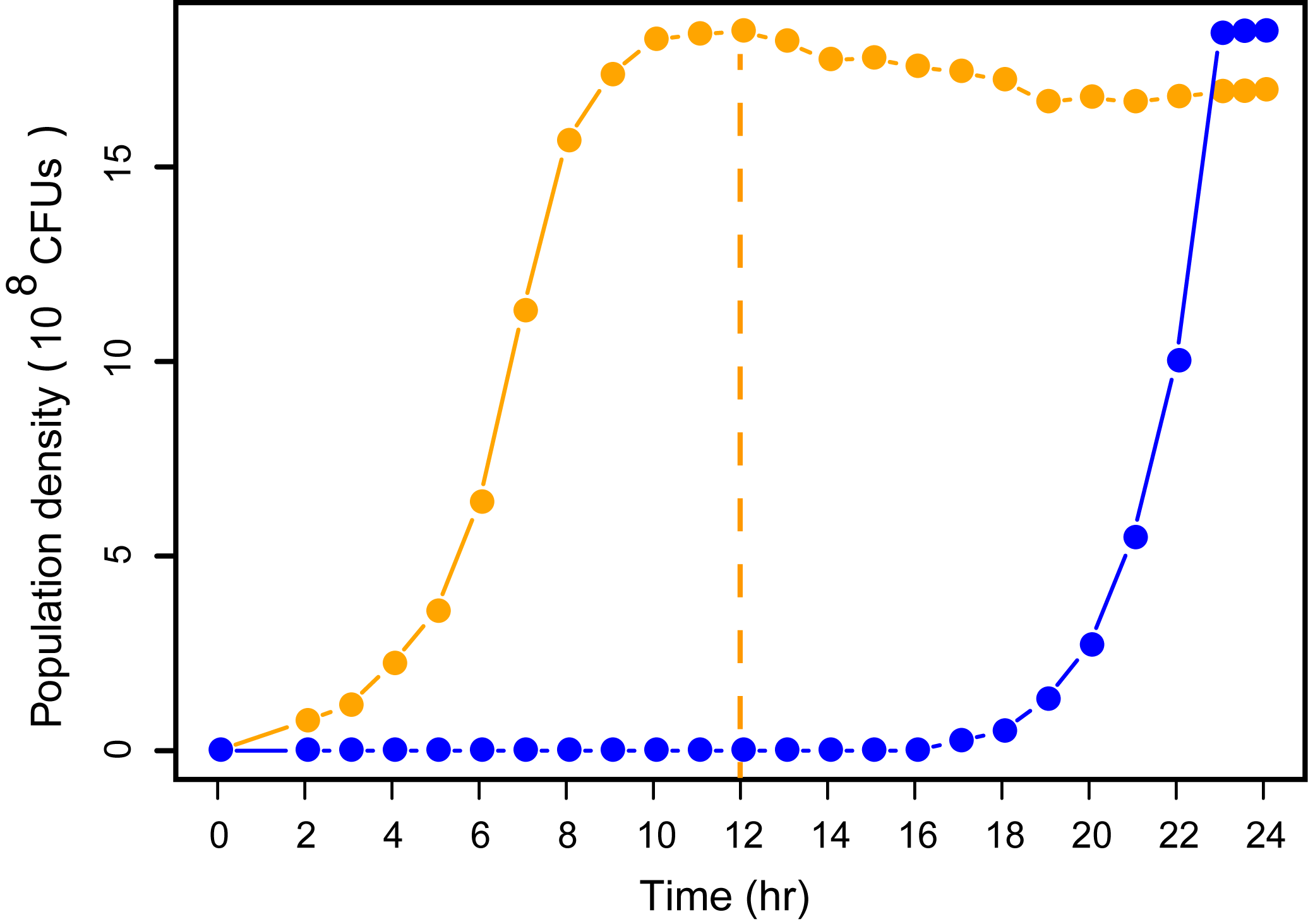

Supplement: Figure S1 — The growth curve of Keio progenitor BW25113 in the High and Low treatments over a 24-h cycle. The orange vertical line indicates the timing of transfer to next growth cycle in large populations. Orange: High treatment; blue: Low treatment. (TIF) [file pone.0105369.s001.tif]

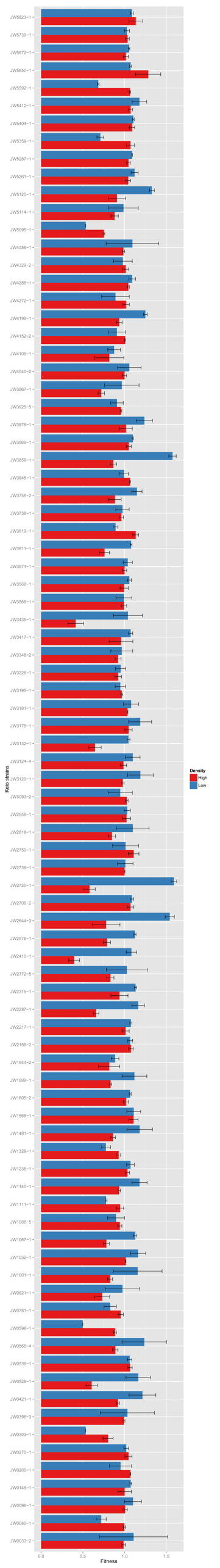

Supplement: Figure S2 — The fitness of all 87 Keio strains in the High and Low treatments. Error bars = 1 SD. (TIF) [file pone.0105369.s002.tif]

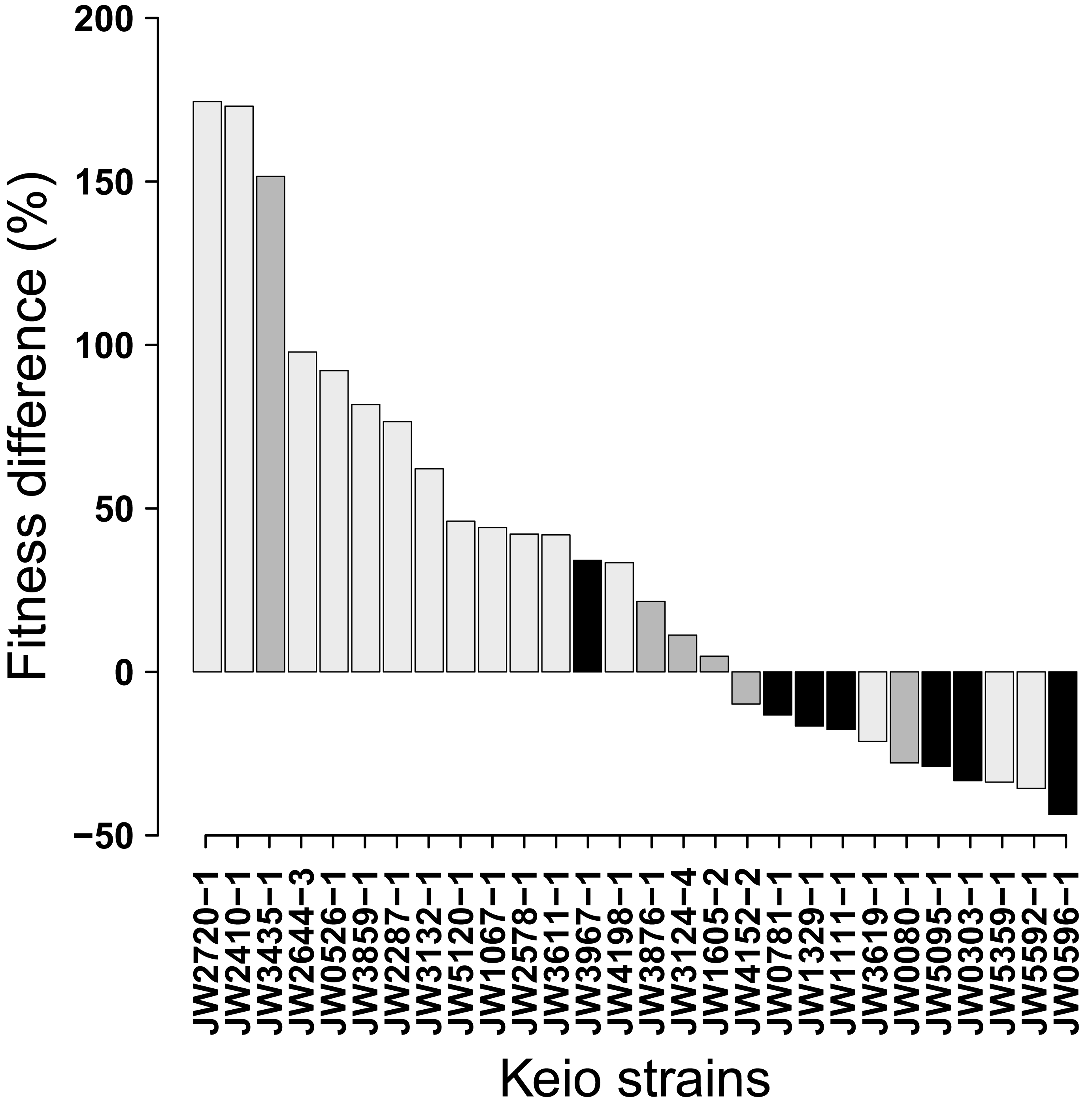

Supplement: Figure S3 — The fitness change between the High and Low treatments in the 28 treatment-responsive Keio strains. Gray bars: strains with deletions exhibiting opposing responses between the High and Low treatments; white bars: strains with deletions neutral in one treatment but non-neutral in the other; black bars: strains with deletions non-neutral in the same direction in both treatments. (TIF) [file pone.0105369.s003.tif]
