# Supplementary material for: Variation in the Fitness Effects of Mutations with Population Density and Size in Escherichia coli
Source: PLoS One. 2014 Aug 14;9(8):e105369. doi: 10.1371/journal.pone.0105369 (PMC4133409; doi:10.1371/journal.pone.0105369)
Supplement: Table S1 — The 100 Keio strains used in this study. (DOCX) [file pone.0105369.s004.docx]

**Table S1**. The 100 Keio strains used in this study.

| JW ID | Gene | Locus | COG ID^a^ | COG code^b^ | CGSC# |
| --- | --- | --- | --- | --- | --- |
| JW0033-2 | *caiF* | b0034 | - | K | 8350 |
| JW0080-1 | *mraW* | b0082 | COG0275 | M | 8380 |
| JW0099-1 | *yacF* | b0102 | COG4582 | S | 8381 |
| JW0148-1 | *fhuD* | b0152 | COG0614 | P | 8418 |
| JW0200-1 | *yafE* | b0210 | COG0500 | Q | 8441 |
| JW0200-1 | *yafE* | b0210 | COG0500 | R | 8441 |
| JW0200-1 | *yafE* | b0242 | COG0263 | E | 8441 |
| JW0232-1 | *proB* | b0276 | - | R | 8467 |
| JW0270-1 | *yagJ* | b0311 | COG2303 | E | 8483 |
| JW0303-1 | *betA* | b0311 | COG2303 | N | 8503 |
| JW0396-3 | *tgt* | b0406 | COG0343 | J | 8571 |
| JW0421-1 | *cyoB* | b0431 | COG0843 | C | 8585 |
| JW0526-1 | *ybcC* | b0539 | - | V | 8662 |
| JW0536-1 | *ninE* | b0548 | - | R | 8669 |
| JW0565-4 | *pheP* | b0576 | COG1113 | E | 8690 |
| JW0596-1 | *ybdO* | b0603 | COG0583 | K | 8712 |
| JW0732-1 | *ybgF* | b0742 | COG1729 | S | 11617 |
| JW0774-1^c^ | *ybhQ* | b0791 | - | M | 11712 |
| JW0781-1 | *rhlE* | b0797 | COG0513 | J | 8834 |
| JW0781-1 | *rhlE* | b0797 | COG0513 | K | 8834 |
| JW0781-1 | *rhlE* | b0797 | COG0513 | L | 8834 |
| JW0821-1 | *yliI* | b0837 | COG2133 | G | 8861 |
| JW0891-1^c^ | *aroA* | b0908 | COG0128 | E | 8914 |
| JW1001-1 | *putP* | b1015 | COG0591 | E | 8982 |
| JW1001-1 | *putP* | b1015 | COG0591 | H | 8982 |
| JW1001-1 | *putP* | b1015 | COG0591 | R | 8982 |
| JW1032-1 | *ymdB* | b1045 | COG2110 | R | 8998 |
| JW1032-1 | *ymdB* | b1045 | COG2110 | S | 8998 |
| JW1067-1 | *flgI* | b1080 | - | N | 9023 |
| JW1088-5 | *fhuE* | b1102 | COG4773 | P | 9032 |
| JW1111-1 | *potB* | b1125 | COG1176 | E | 9042 |
| JW1122-2^c^ | *icd* | b1136 | COG0538 | C | 9051 |
| JW1140-1 | *ycfK* | b1154 | - | R | 9062 |
| JW1235-1 | *oppA* | b1243 | COG4166 | E | 9114 |
| JW1327-1 | *uspE* | b1333 | COG0589 | T | 9186 |
| JW1329-1 | *ogt* | b1335 | COG0350 | L | 9188 |
| JW1481-1 | *ddpB* | b1486 | COG0601 | E | 9287 |
| JW1481-1 | *ddpB* | b1486 | COG0601 | P | 9287 |
| JW1568-1 | *ydfE* | b1577 | - | R | 11817 |
| JW1605-2 | *manA* | b1613 | COG1482 | G | 9366 |
| JW1849-2 | *ruvB* | b1860 | COG2255 | L | 9543 |
| JW1889-1 | *araF* | b1901 | COG1879 | G | 9571 |
| JW1944-2 | *dcm* | b1961 | COG0270 | L | 9617 |
| JW2188-2 | *ccmB* | b2200 | COG2386 | O | 9770 |
| JW2217-1 | *atoE* | b2223 | COG2031 | I | 9788 |
| JW2287-1 | *yfbQ* | b2290 | COG0436 | E | 9839 |
| JW2317-2^c^ | *pdxB* | b2320 | COG0111 | E | 9859 |
| JW2317-2^c^ | *pdxB* | b2320 | COG0111 | H | 9859 |
| JW2319-1 | *yfcJ* | b2322 | COG0477 | E | 9861 |
| JW2319-1 | *yfcJ* | b2322 | COG0477 | G | 9861 |
| JW2319-1 | *yfcJ* | b2322 | COG0477 | P | 9861 |
| JW2319-1 | *yfcJ* | b2322 | COG0477 | R | 9861 |
| JW2372-5 | *yfdX* | b2375 | - | S | 9895 |
| JW2410-1 | *crr* | b2417 | COG2190 | G | 9919 |
| JW2578-1 | *yfiA* | b2597 | COG1544 | J | 10046 |
| JW2644-3 | *stpA* | b2669 | COG2916 | R | 10083 |
| JW2706-2 | *ygbJ* | b2736 | COG2084 | I | 10129 |
| JW2720-1 | *cysC* | b2750 | COG0529 | P | 10139 |
| JW2738-1 | *ygcP* | b2768 | COG1954 | K | 10154 |
| JW2759-1 | *gudX* | b2788 | COG4948 | M | 10162 |
| JW2759-1 | *gudX* | b2788 | COG4948 | R | 10162 |
| JW2818-1 | *ygeF* | b2850 | - | R | 10200 |
| JW2958-1 | *hybG* | b2990 | COG0298 | O | 11883 |
| JW3093-2 | *garK* | b3124 | COG1929 | G | 10356 |
| JW3120-1 | *yraQ* | b3151 | COG0701 | R | 10377 |
| JW3124-4 | *yhbQ* | b3155 | COG2827 | L | 10379 |
| JW3132-1 | *nlpI* | b3163 | COG4785 | R | 10385 |
| JW3140-5^c^ | *argG* | b3172 | COG0137 | E | 8306 |
| JW3178-1 | *yhcC* | b3211 | COG1242 | R | 11828 |
| JW3181-1 | *gltF* | b3214 | - | K | 10413 |
| JW3195-1 | *nanR* | b3226 | COG2186 | K | 10424 |
| JW3226-1 | *panF* | b3258 | COG4145 | H | 10440 |
| JW3348-2 | *gph* | b3385 | COG0546 | R | 10498 |
| JW3417-1 | *ugpA* | b3452 | COG1175 | G | 10541 |
| JW3435-1 | *yhhP* | b3470 | COG0425 | O | 10552 |
| JW3496-1 | *dctA* | b3528 | COG1301 | C | 10589 |
| JW3566-1 | *rhsA* | b3593 | COG3209 | M | 10629 |
| JW3568-1 | *yibA* | b3594 | COG1413 | C | 10630 |
| JW3574-1 | *mtlD* | b3600 | COG0246 | G | 10634 |
| JW3611-1 | *rpmG* | b3636 | COG0267 | J | 10657 |
| JW3619-1 | *yicC* | b3644 | COG1561 | S | 10660 |
| JW3738-1 | *yifB* | b3765 | COG0606 | O | 10728 |
| JW3747-2^c^ | *ilvC* | b3774 | COG0059 | E | 10735 |
| JW3747-2^c^ | *ilvC* | b3774 | COG0059 | H | 10735 |
| JW3758-2 | *rfe* | b3784 | COG0472 | M | 10741 |
| JW3841-1^c^ | *glnA* | b3870 | COG0174 | E | 10775 |
| JW3845-1 | *yihN* | b3874 | COG0477 | E | 10778 |
| JW3845-1 | *yihN* | b3874 | COG0477 | G | 10778 |
| JW3845-1 | *yihN* | b3874 | COG0477 | P | 10778 |
| JW3845-1 | *yihN* | b3874 | COG0477 | R | 10778 |
| JW3859-1 | *yiiD* | b3888 | COG0454 | K | 10787 |
| JW3859-1 | *yiiD* | b3888 | COG0454 | R | 10787 |
| JW3869-1 | *frvX* | b3898 | COG1363 | G | 10794 |
| JW3876-1 | *rhaS* | b3905 | COG2207 | K | 11807 |
| JW3925-5 | *frwD* | b3953 | COG1445 | G | 10834 |
| JW3967-1 | *zraS* | b4003 | COG0642 | T | 10853 |
| JW4040-2 | *fdhF* | b4079 | COG0243 | C | 10908 |
| JW4108-1 | *ecnB* | b4411 | COG5510 | S | 10958 |
| JW4122-3 | *rsgA* | b4161 | COG1162 | R | 10968 |
| JW4152-2 | *ulaB* | b4194 | COG3414 | G | 10989 |
| JW4198-1 | *treC* | b4239 | COG0366 | G | 11021 |
| JW4272-1 | *yjhS* | b4309 | COG2801 | L | 11835 |
| JW4286-1 | *uxuB* | b4323 | COG0246 | G | 11071 |
| JW4329-2 | *yjjQ* | b4365 | COG2197 | K | 11088 |
| JW4329-2 | *yjjQ* | b4365 | COG2197 | T | 11088 |
| JW4358-1 | *ytjC* | b4395 | COG0406 | G | 11111 |
| JW5095-1 | *ybfH* | b0691 | - | S | 12198 |
| JW5114-1 | *ybjK* | b0846 | COG3226 | S | 11183 |
| JW5120-1 | *ycaI* | b0913 | COG0658 | R | 11188 |
| JW5120-1 | ycaI | b0913 | COG2333 | R | 11188 |
| JW5261-1 | *ynfH* | b1590 | COG3302 | R | 11277 |
| JW5287-1 | *ynjE* | b1757 | COG2897 | P | 11294 |
| JW5359-1 | *yeiS* | b2145 | - | M | 11340 |
| JW5363-1^c^ | *bcr* | b2182 | COG0477 | E | 11343 |
| JW5363-1^c^ | *bcr* | b2182 | COG0477 | G | 11343 |
| JW5363-1^c^ | *bcr* | b2182 | COG0477 | P | 11343 |
| JW5363-1^c^ | *bcr* | b2182 | COG0477 | R | 11343 |
| JW5404-1 | *sseB* | b2522 | - | R | 11368 |
| JW5412-1 | *yfiL* | b2602 | - | V | 11374 |
| JW5592-1 | *dapF* | b3809 | COG0253 | E | 11481 |
| JW5650-1 | *sgbU* | b3582 | COG3623 | G | 11500 |
| JW5672-1 | *yhiQ* | b3497 | COG0500 | Q | 11511 |
| JW5672-1 | *yhiQ* | b3497 | COG0500 | R | 11511 |
| JW5739-1 | *yjeM* | b4156 | COG0531 | E | 11549 |

^a^ -: no COG ID

^b^ some genes have more than one function

^c^ not included in fitness assays due to slow growth during conditioning
