# Supplementary material for: Variation in the Fitness Effects of Mutations with Population Density and Size in Escherichia coli
Source: PLoS One. 2014 Aug 14;9(8):e105369. doi: 10.1371/journal.pone.0105369 (PMC4133409; doi:10.1371/journal.pone.0105369)
Supplement: Table S2 — The functions of the deleted genes in the Keio strains showing population density-dependent fitness difference. (DOCX) [file pone.0105369.s005.docx]

**Table S2.** The functions of the deleted genes in the Keio strains showing population density-dependent fitness difference.

| Functional Category | COG Code | JW ID | Gene^a^ |
| --- | --- | --- | --- |
| *Information storage and processing* |  |  |  |
| Translation, ribosomal structure and biogenesis | J | JW2578-1 | *yfiA* |
|  |  | JW3611-1 | *rpmG* |
|  |  | JW0781-1 | *rhlE* |
| Transcription | K | JW0596-1 | *ybdO* |
|  |  | JW2644-3 | *stpA* |
|  |  | JW0781-1 | *rhlE* |
|  |  | JW3876-1 | *rhaS* |
| Replication, recombination and repair | L | JW1329-1 | *ogt* |
|  |  | JW0781-1 | *rhlE* |
| *Cellular processes and signaling* |  |  |  |
| Cell cycle control, cell division, chromosome partitioning | D | JW3132-1 | *nlpI* |
| Cell wall/membrane/envelope biogenesis | M | JW0080-1 | *mraW* |
|  |  | JW5359-1 | *yeiS* |
| Cell motility | N | JW0303-1 | *betA* |
|  |  | JW1067-1 | *flgI* |
| Posttranslational modification, protein turnover, chaperones | O | JW3435-1 | *yhhP* |
| Signal transduction mechanisms | T | JW3967-1 | *zraS* |
| Defense mechanisms | V | JW0526-1 | *ybcC* |
| *Metabolism* |  |  |  |
| Carbohydrate transport and metabolism | G | JW1605-2 | *manA* |
|  |  | JW2410-1 | *crr* |
|  |  | JW4152-2 | *ulaB* |
|  |  | JW4198-1 | *treC* |
|  |  | JW5592-1 | *dapF* |
| Amino acid transport and metabolism | E | JW1111-1 | *potB* |
|  |  | JW2287-1 | *yfbQ* |
|  |  | JW0303-1 | *betA* |
| Inorganic ion transport and metabolism | P | JW2720-1 | *cysC* |
| *Poorly characterized* |  |  |  |
| Function unknown | S | JW3124-4 | *yhbQ* |
|  |  | JW3619-1 | *yicC* |
|  |  | JW3859-1 | *yiiD* |
|  |  | JW5095-1 | *ybfH* |
|  |  | JW5120-1 | *ycaI* |

^a^ *betA* was dual functional (EN) and *rhlE* triple functional (JKL)
